# Heterogeneity of Glucose Transport in Lung Cancer

**DOI:** 10.3390/biom10060868

**Published:** 2020-06-05

**Authors:** Cesar A. Martinez, Claudio Scafoglio

**Affiliations:** Division of Pulmonary and Critical Care Medicine, David Geffen School of Medicine, University of California Los Angeles, Los Angeles, CA 90095, USA; CesarAMartinez@mednet.ucla.edu

**Keywords:** glucose metabolism, glucose transport, cancer, lung cancer, tumor heterogeneity

## Abstract

Increased glucose uptake is a known hallmark of cancer. Cancer cells need glucose for energy production via glycolysis and the tricarboxylic acid cycle, and also to fuel the pentose phosphate pathway, the serine biosynthetic pathway, lipogenesis, and the hexosamine pathway. For this reason, glucose transport inhibition is an emerging new treatment for different malignancies, including lung cancer. However, studies both in animal models and in humans have shown high levels of heterogeneity in the utilization of glucose and other metabolites in cancer, unveiling a complexity that is difficult to target therapeutically. Here, we present an overview of different levels of heterogeneity in glucose uptake and utilization in lung cancer, with diagnostic and therapeutic implications.

## 1. Glucose Metabolism in Cancer

Increased glucose uptake is a hallmark of cancer. The increased metabolic requirements of neoplastic cells have been extensively studied, and therapeutic strategies are being investigated to hinder tumor growth by interfering with glucose uptake and metabolism in cancer cells [1,2,3,4,5]. The original observations by Otto Warburg that cancers utilized glucose anaerobically even in the presence of oxygen were consistent with increased requirement of glucose [6]. The conversion of glucose to lactate yields only 2 moles of ATP per mole of glucose, whereas mitochondrial oxidative phosphorylation results in the net production of 30 moles of ATP per mole of glucose [7]. Warburg concluded that a mitochondrial dysfunction was at the origin of cellular transformation, and to compensate for the inefficient respiration, the transformed cell needed to increase its glucose uptake and fermentation to sustain the metabolic requirements of the cell [6].

However, more recent observations have shown that mitochondrial activity is not impaired in cancer cells [8] and is indeed required for neoplastic transformation [9]. The reduced mitochondrial activity observed by Warburg in cancer cells could have been induced by the Crabtree effect, i.e., high rates of glucose uptake and glycolysis in cancer cells can inhibit mitochondrial respiration, likely due to competition between glycolysis and oxidative phosphorylation for ADP and inorganic phosphate [10]. This leaves open the question of why cancer cells need to increase their glucose uptake and glycolytic metabolism even in the presence of oxygen. Although a definitive answer to this question has not been provided, several lines of evidence have suggested that glycolysis is preferred to mitochondrial respiration in rapidly proliferating tissues, in the context of neoplastic transformation, and also in normal physiological processes, such as embryonic development [11,12], reprogramming of induced pluripotent stem cells [13], and immune cell activation [14,15]. Since the cellular respiratory machinery is easily saturated, especially in conditions of low oxygen availability that are frequent in cancer, aerobic glycolysis provides a low-yield, but a high-rate alternative to produce large quantities of ATP in short periods of time [16]. Increased glucose uptake and slowed mitochondrial utilization of glucose in rapidly proliferating tissues provides a fast source of energy through ATP, and also feeds a number of anabolic pathways that are required for buildup of biomass, including the pentose phosphate pathway, the serine biosynthetic pathway, the hexosamine biosynthetic pathway, and lipid biosynthesis.

**Figure 1:**
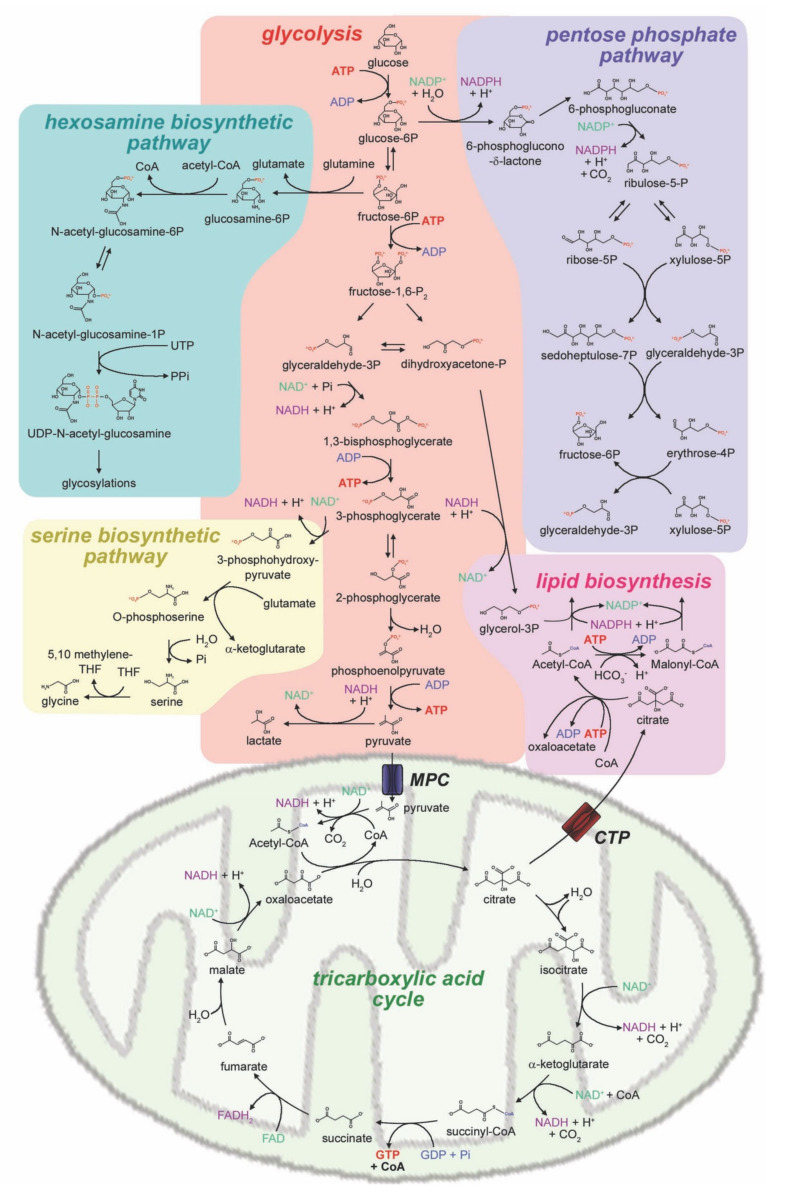
Metabolic pathways of glucose utilization in cancer cells.

Figure 1 summarizes the major metabolic pathways branching out of glucose metabolism. Glycolysis catabolizes glucose to pyruvate, with production of ATP and reduction of 2 moles of NAD+ to NADH per mole of glucose. Then, pyruvate can feed into the aerobic respiration or be converted anaerobically to lactate. In normal tissues, pyruvate is transported into mitochondria by the mitochondrial pyruvate carrier (MPC); the pyruvate dehydrogenase complex catalyzes oxidative decarboxylation into acetyl-coenzyme A (CoA). Acetyl-CoA can feed the tricarboxylic acid cycle and the mitochondrial electron transport chain to produce energy. Alternatively, pyruvate can be reduced to lactate by lactate dehydrogenase. This reaction defines the anaerobic utilization of glucose, typical of the Warburg effect. Lactic acid can be exported by the cell by monocarboxylate transporters (MCT) [17]. The lactate transporters most frequently overexpressed in cancer, MCT1 and MCT4, are attractive therapeutic targets to interfere with the Warburg effect [18,19,20]. The increased production and export of lactate in cancer cells have the following three important consequences: (1) the reduction of pyruvate to lactate utilizes NADH, returning NAD+ to sustain the increased rate of glycolysis in cancer cells; (2) export of lactate and H^+^ ions acidifies the tumor microenvironment, reducing the viability of normal cells and favoring the infiltration of neoplastic cells in normal tissues [21] and angiogenesis [22]; and (3) lactate acts as an autocrine and paracrine factor by affecting pH and metabolism, and also by binding specific receptors on target cells, GPR81, and activating a signaling cascade with activation of protein kinase A [23]. GPR81 on cancer cells stimulates lactate uptake, mitochondrial metabolism, angiogenesis, and tumor growth [24,25].

The pentose phosphate pathway is an alternative route of glucose utilization that results in the production of NADPH, ribose, and glycolytic intermediates [26]. The pentose phosphate pathway comprises an oxidative and a non-oxidative branch. The oxidative branch is initiated by conversion of glucose-6-phosphate to 6-phosphogluconate by glucose-6-phosphate dehydrogenase and culminates in the production of ribose-5P and NADPH [26]. Ribose-5P is used for de novo nucleotide biosynthesis, providing the building blocks of DNA replication for cell proliferation. NADPH is necessary to provide reducing equivalents used for biosynthetic processes (fatty acid synthesis, nucleotide synthesis), and also to re-equilibrate the balance between oxidized and reduced glutathione in conditions of oxidative stress. The non-oxidative phase of the pentose phosphate pathway can feed back into glycolysis through the production of fructose-6-phosphate and glyceraldehyde-3-phosphate [26]. The pentose phosphate pathway is upregulated in cancer and has been proposed as a therapeutic target [27].

The serine biosynthetic pathway produces the amino acid serine from the glycolysis intermediate 3-phosphoglycerate via the enzyme phosphoglycerate dehydrogenase [28]. Serine is a major contributor to the one-carbon pool, as it is converted to glycine donating a methyl group to N5,N10-methylenetetrahydrofolate. This is required for purine biosynthesis, essential for DNA synthesis in rapidly proliferating cells. Upregulated de novo serine biosynthesis is common in cancer and supports tumor growth [29,30]. Glycine is also required for glutathione synthesis, contributing to the maintenance of cellular redox balance [31].

In the hexosamine biosynthetic pathway, an aminotransferase reaction commits fructose-6-phosphate from glycolysis to the production of N-acetyl-glucosamine for glycosylation reactions. This pathway has been described as a sensor of nutrient availability that stimulates cancer cell growth and proliferation when the abundance of glucose and other nutrients in the microenvironment allow it [32]. The hexosamine biosynthetic pathway is required for Kras-induced transformation [33], and increased hexosamine biosynthetic pathway has been associated with epithelial to mesenchymal transition [34], cancer stem cell phenotype [35], and regulates the function of receptor tyrosine kinases [36] and oncogenes [33].

De novo lipid biosynthesis occurs when citrate produced in the TCA cycle is exported from the mitochondria by the citrate transport protein (CTP). In the cytoplasm, citrate is converted into acetyl-CoA via ATP citrate lyase. The subsequent carboxylation of acetyl-CoA commits the resulting malonyl-CoA to fatty acid biosynthesis. The glycolysis intermediate dihydroxyacetone-phosphate provides glycerol for the synthesis of triglycerides and phospholipids. Although lipids can be acquired from the host, several studies have confirmed that cancer cells rely heavily on de novo lipid biosynthesis for cell growth, proliferation, energy source, and signaling [37,38].

The increased glycolysis and the reduced utilization of pyruvate in the mitochondria create a bottleneck effect enriching the cells of metabolites required for the described branching anabolic and redox pathways. This effect is achieved in many cancers by upregulation of the fetal isoform of pyruvate kinase, M2, which has slower and more regulated activity than the adult isoform M1, thus slowing down the last step of glycolysis [39]. Strategies to increase the activity of PKM2 are expected to reverse the Warburg effect and suppress tumorigenesis [40]. The increased glycolytic flux also results in accumulation of NADH, which needs to be reduced to NAD+ for the cancer cell to maintain a functional glycolytic flux. This is achieved by the increased activity of lactic dehydrogenase, as discussed above.

Despite the importance of aerobic glycolysis in cancer, recent metabolic tracing studies in human cancers have shown that the original Warburg hypothesis that cancer is caused by a mitochondrial defect is inaccurate [41]. Cancers upregulate both glycolysis and mitochondrial metabolism at the same time. This apparent paradox is explained partly by the presence of regulatory mechanisms (such as PKM2 expression) that can switch the utilization of pyruvate between mitochondrial respiration and aerobic glycolysis, according to the metabolic necessity of the cell. In addition, cancers are typically heterogeneous and not all the cells in a tumor have the same metabolic phenotype. This metabolic heterogeneity allows the co-existence of glycolytic and oxidative cells in different regions of the same tumor, as a consequence of a complex interplay between oncogenic alterations and microenvironmental factors. We explore these interactions in the next section.

## 2. Tumor Heterogeneity

**Figure 2:**
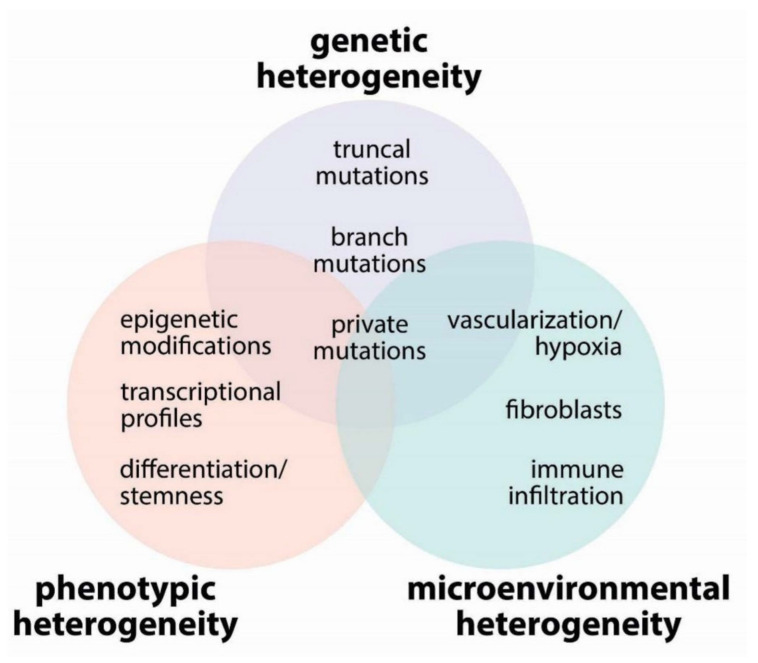
Major determinants of tumor heterogeneity.

Tumor heterogeneity is a well described hallmark of cancer and poses a significant obstacle to effective treatments. There are three major sources of tumor heterogeneity, i.e., genetic, phenotypic, and microenvironmental (Figure 2).

### 2.1. Genetic Heterogeneity

Cancer cells are characterized by major dysfunctions of DNA repair systems and DNA damage checkpoint regulators, leading to increased frequency of mutations and genomic instability [42]. Genetic heterogeneity is a consequence of this genomic instability, which over time causes divergence of subclonal populations of cancer cells [43]. Multiregion sequencing and single-cell sequencing in tumors has shown that not all tumor regions share the same genomic landscape [44,45]. Genetic abnormalities contributing to the aggressiveness of cancers can fall into one of the following: truncal, branch, or private mutations.

Truncal mutations are the initiating mutations that occurr early in the tumorigenic process and are present in all the cells of the tumor mass [45]. These mutations can involve either loss-of-function mutation in tumor suppressor genes or activating mutations in proto-oncogenes that drive the clonal expansion of premalignant cells and sets the stage for progression to invasive cancer. Subsequently, cancers evolve as a complex mixture of heterogeneous neoplastic clones that accumulate branching mutations, detected in some areas of the tumor but not in others, and private mutations, present only in one region [44,45]. While many mutations are passengers with no functional consequences, some of the branching and private mutations confer peculiar phenotypic characters causing divergence of distinct subclonal populations from the original clonal tumor. These clones compete against each other in the tumor microenvironment, and the clonal dynamics of the tumor determines the overall biology and evolution of the neoplasia over time and the sensitivity to therapeutic interventions [43].

### 2.2. Phenotypic Heterogeneity

**Figure 3:**
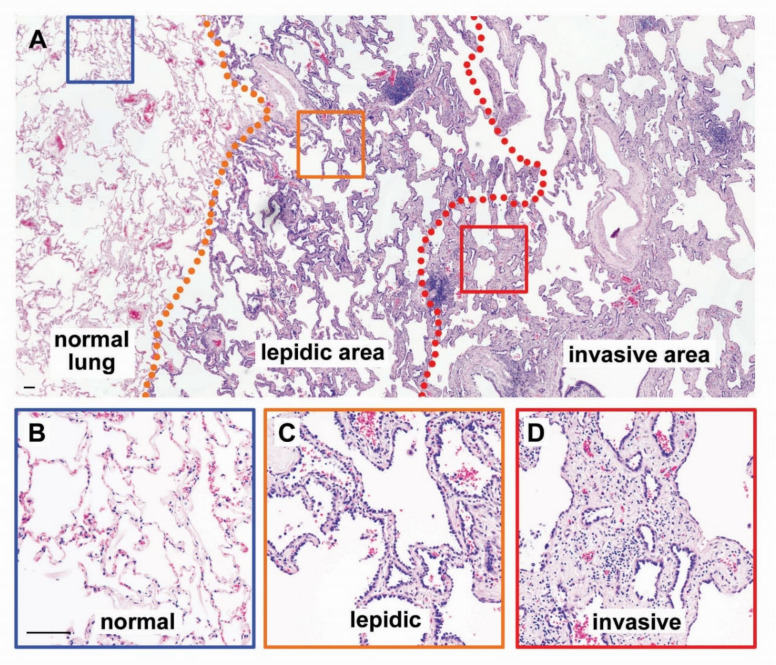
Morphological heterogeneity in lung adenocarcinoma. A representative hematoxylin and eosin stain of human lung adenocarcinoma is presented. (**A**) Low-magnification picture that shows the transition from normal lung tissue to invasive cancer. The orange dotted line delimits the transition from normal tissue to well-differentiated (lepidic) cancer. The red line delimits the transition from well-differentiated (lepidic) to invasive cancer. The areas delimited by the blue, orange, and red squares are shown in the bottom panels at higher magnification; (**B**–**D**) Higher magnifications of the areas of normal lung (**B**), lepidic adenocarcinoma (**C**), and invasive adenocarcinoma (**D**) highlighted by the color-coded squares. Scalebars, 100 µm.

Phenotypic heterogeneity is likely determined by a complex interplay of genetic, epigenetic, and environmental factors. If the subsequent accumulation of different oncogenic mutations in different tumor subclones induces the acquisition of a more and more de-differentiated and stem-like phenotype, then the whole tumor will comprise a heterogeneous mixture of cells whose phenotype is blocked at different steps of tissue differentiation [46]. This phenotypic heterogeneity translates into morphological heterogeneity. For lung adenocarcinomas, which develop from distal airways, premalignant lesions and well-differentiated tumors preserve the air-filled alveolar structures, in a pathologic pattern defined as lepidic growth; this is characterized by proliferation of neoplastic cells along the preformed alveolar wall structures, which become thickened but preserve their structure [47]. When the tumors become invasive, they acquire moderately differentiated morphology, with the formation of acinar or papillary structures. Finally, the poorly differentiated tumors lose any recognizable structures and grow as nests of tightly juxtaposed, non-polarized cells [47]. In lung adenocarcinoma specimens, it is very common to find mixed morphological patterns, with the invasive, less differentiated components typically located in the middle of the tumor mass and the well-differentiated, lepidic component at the periphery, representing a transition zone between the normal lung and the invasive cancer tissues (Figure 3). Since the tumor grade has relevant prognostic value, the guidelines of the International Association for the Study of Lung Cancer recommend that pathologists specify the relative contribution of well-, moderately-, and poorly-differentiated areas to the overall tumor mass [48]. Phenotypic classification has clinically relevant consequences: the five-year disease-free survival of patients with stage I lung adenocarcinoma with lepidic-predominant adenocarcinoma is 94.5%, vs. 85.4%–89.7% of moderately differentiated tumors, and only 54% of solid, poorly-differentiated tumors [49]. Although the relationships among mutational, transcriptional, and morphologic heterogeneity have not been studied, this is an important question in cancer biology with potential clinical implications, since the clonal heterogeneity of cancer cells is considered to be a source of resistance to current anticancer therapies [43].

### 2.3. Microenvironmental Heterogeneity

Microenvironmental heterogeneity refers to the contribution of normal host cells and tissues that react to the neoplastic proliferation, affecting both positively and negatively the carcinogenesis process. These factors include blood vessels, fibroblasts and connective tissue, and immune cells as follows:

#### 2.3.1. Blood Vessels

In normal tissues, each cell needs to be within 100 µm distance from a capillary vessel to receive an adequate supply of nutrients and oxygen [50]. As tumor cells proliferate in an uncontrolled fashion and stretch the spatial boundaries of the host tissues, areas of the tumors are bound to be located at a distance from viable blood vessels that limits the diffusion of oxygen and nutrients from the capillaries, leading to ischemia and necrosis [51]. Hypoxia and oncogenic signals induce the secretion of neoangiogenic factors such as VEGF, which induce neoangiogenesis [52]. However, the neoplastic blood vessels typically develop with an incomplete and disordered architecture [53], causing edema and microhemorrhages, which are accompanied by further blood vessel compression and consequent ischemia, thus, increasing the heterogeneity of the tumor microenvironment. Even if lymphangiogenesis occurs in parallel with neoangiogenesis, the intratumoral lymph vessels are usually not enough to relieve the intratumoral pressures [54]. In this context, tumor cells are exposed to waves of changing nutrient and oxygen gradients, which fluctuate as the tumor perfusion and vascularization evolves. Chronic cycling hypoxia has been proposed as a determining factor for tumor progression and resistance to therapy [55].

#### 2.3.2. Fibroblasts

The cancer tissue has been described as a chronic wound that never heals [56]. The uncontrolled growth of cancer cells poses a constant disruption of the normal tissue architecture that activates a chronic wound healing process, recruiting activated fibroblasts in the tumor microenvironment. Cancer-associated fibroblasts are activated by cytokines secreted by tumor cells, including TGF-β [57] and PDGF [58], and paradoxically, by immune cells recruited to mount a response against cancer cells. IL-1β and IL-6 secretion by cancer cells or by immune cells induces activation of NF-kB transcriptional targets in fibroblasts, activating a pro-tumorigenic program [59,60]. Activated fibroblasts perform a series of functions that support the growth of tumor cells, such as remodeling of the extracellular matrix to promote cancer cell migration [61], recruitment of inflammatory and immune cells [62], stimulation of angiogenesis [63], secretion of growth factors [64], and cancer stem cell niche [64].

#### 2.3.3. Immune Cells

The immune system plays a major role in the carcinogenic process. The immune system includes an innate and an adaptive branch. The innate branch constitutes the first defense against pathogens, and is activated by pattern recognition receptors which bind conserved microbial determinants. The adaptive branch requires specific identification of pathogen antigens by immune cells and the mounting of a stronger response and immunological memory. The immune response against cancer is very complex and involves several cell types cross talking with each other [65]. For simplicity, we will focus on two major players of the immune response against cancer, i.e., macrophages and T cells.

Inflammation, which relies mostly on cells of the innate immune system, is both a promoting factor associated with carcinogenic stimuli before the development of cancer and a first response of the normal tissue to the expansion of incipient cancers [65]. Macrophages and neutrophils represent the first responders to exogenous threats such as bacteria and viruses. Macrophages can display a tumor-suppressing classical activation phenotype (M1), producing antitumor factors including tumor necrosis factor alpha, reactive oxygen species, and nitric oxide [66]. Chronic inflammation associated with cancer is characterized by an alternative activation (M2) of macrophages, which promotes tissue repair and cancer progression by suppressing cytotoxic T-cell anticancer response, stimulating angiogenesis and tumor cell migration, and producing growth factors and matrix proteases that promote invasion [66].

As cancer progresses and accumulates multiple mutations, chromosomal aberrations and genomic instability, neoplastic cells present neoantigens carried by class I major histocompatibility complex (MHC) proteins [67]. Recognition of these epitopes as non-self, allows for targeting of cancer cells by cytotoxic CD8+ lymphocytes. Consistently, the presence of infiltrating T cells in the tumor is a positive prognostic factor in cancer [68]. However, cancer cells deploy a number of strategies to evade immune surveillance, such as the loss of class I MHC, downregulation of neoantigens, alteration of cell death signaling, production of immuno-suppressive cytokines, and expression of immune checkpoint receptors such as PD-L1 and CTLA4, which induce T cell exhaustion and activation of suppressor T cells [69]. Therefore, tumor evolution is characterized by a constant dynamical interplay between immune defense against cancer invasion and mechanisms of cancer cell escape. In the context of cancer heterogeneity, the immune system applies an additional selection pressure to cancer cells, eliminating the most immunogenic cancer clones, and thus contributing to mold the evolution of cancer phenotype in a process called immunoediting [70].

## 3. Metabolic Interactions in the Tumor Microenvironment

**Figure 4:**
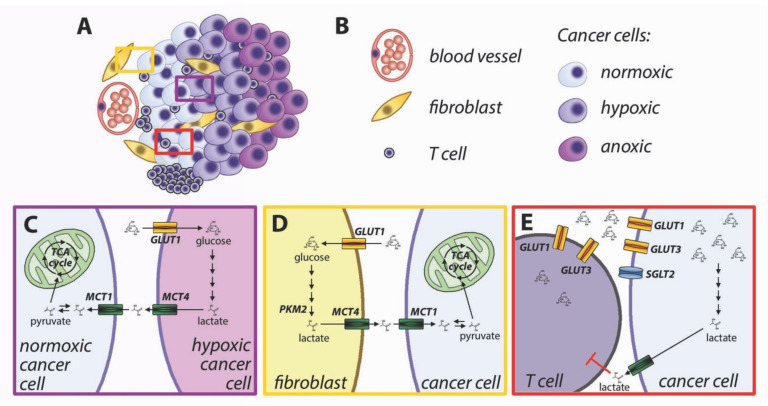
Metabolic interactions in the tumor microenvironment. (**A**) Schematic representation of the tumor microenvironment; (**B**) Description of the different cell types presented in (A); (**C**–**E**) Color-coded higher magnifications of the rectangles presented in (A), highlighting metabolic interactions in the tumor microenvironment. (**C**) lactate-fueled respiration, (**D**) reverse Warburg effect and (**E**) immune metabolic competition.

Given the complexity and the regional heterogeneity of the tumor microenvironment, it is not surprising that complex metabolic interactions occur in cancer tissues. Here, we review three major mechanisms of metabolic cooperation or competition in the tumor microenvironment. These include lactate-fueled glycolysis, reverse Warburg effect, and immune metabolic competition and are described as follows:

(1) Lactate-fueled respiration [71]. Metabolic tracing studies in humans have shown that tumors take up not only glucose but also lactate from the bloodstream, and the FDG-avid tumors are also responsible for higher lactate uptake [72]. This apparent paradox is consistent with lactate-fueled respiration, which is a consequence of the microenvironmental heterogeneity of most solid tumors. The neoplastic tissues are characterized by steep gradients of oxygen and nutrients (Figure 4A,B), and the metabolism of well-perfused cells is different from that of poorly perfused cells [73]. Hypoxia induces glucose uptake and increased glycolysis via activation of hypoxia-inducible factors (HIFs), which directly induce the transcription of glucose transporter GLUT1 and glycolytic enzymes [74]. Hypoxic cells engage in anaerobic glycolysis and export lactate via upregulation of monocarboxylate transporter MCT4 [75]. MCT4 is adapted to export lactate from cancer cells [76], and its lower affinity for pyruvate prevents an efflux of this metabolite [19], which would hinder the restoration of intracellular NAD+ in highly glycolytic cells. Lactate accumulates in the tumor microenvironment and can be taken up by well-perfused cancer cells via MCT1 [71]. Lactate can be oxidized to pyruvate with the concurrent reduction of NAD+ to NADH. Both pyruvate and NADH can feed the mitochondrial TCA cycle and oxidative phosphorylation (Figure 4C). Cytoplasmic NADH can be transported to the mitochondrial matrix via the malate-aspartate shuttle.
(2) Reverse Warburg effect [77]. Similar to the lactate-fueled glycolysis described in the previous paragraph, the transfer of catabolites from stromal cells can allow tumor cells to replenish their ATP stores. Activated fibroblasts undergo metabolic reprogramming and perform aerobic glycolysis similarly to cancer cells [78]. Loss of caveolin-1 in breast cancer-associated fibroblasts causes oxidative stress leading to HIF-1 activation and induction of glycolysis [77,79]. Oxidative stress in cancer-associated fibroblasts can be caused by hydrogen peroxide produced by cancer cells [80]. Aerobic glycolysis can also be induced in cancer-associated fibroblasts by reduced isocitrate dehydrogenase activity, with a reduction of alpha-ketoglutarate and stabilization of HIF-1 [81]. Direct cell-to-cell contact induces GLUT1 and glycolytic activity in fibroblasts, along with overexpression of MCT4, responsible for cellular export of lactate [82]. The lactate exported by fibroblasts can be imported into cancer cells via MCT1 [82]. Pyruvate can be used by well perfused cancer cells to fuel mitochondrial TCA cycle (Figure 4D).
(3) Immune metabolic competition. The immune responses against cancer cells are shaped by complex interactions between tumor cells, immune cells, and the microenvironment [70]. T cell activation is strictly dependent upon glucose availability [83]. Upon activation, T cells undergo a metabolic switch with increased rate of glucose uptake and utilization [84]. Glucose is imported in T lymphocytes though GLUT3 and GLUT1 transporters. The increased requirement for glucose during T cell activation is supported by upregulation and membrane translocation of GLUT1 [84,85]. Glucose availability is a limiting factor for T cell proliferation [15], cytokine production [83], and cytotoxic activity by CD8+ lymphocytes [86]. The tumor microenvironment is characterized by a lower glucose concentration than normal tissues [87], for insufficient vascularization and increased glucose utilization by cancer cells. Because both cancer cells and lymphocytes display increased reliance on glucose uptake, there is metabolic competition for limiting amounts of glucose in the tumor microenvironment [88,89]. In addition, increased glycolysis in cancer cells produces lactate and acidifies the tumor microenvironment, leading to further inhibition of T cell activation [90,91] (Figure 4E). Comparison of glucose uptake and gene expression patterns in human tumors has suggested that the tumor-specific immunity is hindered in highly glycolytic tumors [92,93]. Inhibition of metabolic reprogramming in cancer cells has been proposed to improve antitumor immunity [94].

The lactate excreted by glycolytic cancer cells also affects the function of tumor-associated macrophages. Macrophages also compete with cancer cells for uptake of glucose. Tumor-associated macrophages show metabolic reprogramming and aerobic glycolysis [95,96]. Lactate activates HIF1 in macrophages inducing a pro-tumorigenic M2 phenotype [97].

In addition to local metabolic interactions in the tumor microenvironment, cancer cells engage in systemic metabolic interactions that support cancer growth. Metabolic tracing studies have shown that cancer cells take up lactic acid from neighboring epithelial and stromal cells, and also from the circulating pool [72,98]. Lactate produced by the muscle or other tissues is a major source of carbon for the TCA cycle in cancer cells [98]. In addition, lactate produced by cancer cells can be utilized by the liver for gluconeogenesis, similar to the well described Cori cycle; in the context of cancer, this is considered to be a futile metabolic cycle that contributes to tumor cachexia [99].

## 4. Heterogeneity of Glucose Transporters in Lung Cancer

Given the increased metabolic needs of cancer cells, glucose transport is often the first limiting step of glucose metabolism in cancer cells [1,100,101,102]. Glucose transporters are upregulated in many cancers and their regulation plays a prominent role in the oncogenic transformation and progression. There are three major classes of glucose transporters as follows: facilitative glucose transporters (GLUTs), sodium-glucose co-transporters (SGLTs), and transporters of the SWEET family. This latter class is largely represented in plants, and only one member is present in the human genome (SWEET1) and is likely involved in the intestinal glucose absorption. Its involvement in cancer is currently unknown.

The physiological features of glucose transporters are summarized in Table 1.

### 4.1. GLUTs and Cancer

The GLUT family (gene family name, SLC2A) includes 14 known transporters with 12 transmembrane domains organized in a pore structure responsible for hexose transport, intracellular N- and C-termini, and two loops, one intracellular and one extracellular [162]. GLUTs are divided into three classes according to their structure and substrate affinity. Class I includes GLUT1–4 and GLUT14, characterized by an extracellular loop between helices 1 and 2 and the intracellular loop between helices 6 and 7. These are the most characterized glucose transporters, with high affinity for glucose and 2-deoxyglyucose and inhibited by cytochalasin B [162,163]. Class II GLUTs, the “odd transporters”, include GLUT5, -7, -9, and -11. They have a general structure that is similar to the class I transporters, but critical residue differences in helix 7 change the substrate specificity of these transporters; class II GLUTs transport fructose with high affinity, can transport glucose (except for GLUT5), but none of them transport 2-deoxy-glucose nor are they inhibited by cytochalasin B [162,163]. Class III GLUTs, the “even transporters”, include GLUT6, -8, -10, -12, and -13. They have the intracellular loop between helices 6 and 7, and the extracellular glycosylated loop between helices 9 and 10. These transporters are less well characterized; they can transport both glucose and fructose, as well as other exoses. Many of these transporters are characterized by an N-terminal dileucine motif responsible for an intracellular, lysosomal, or Golgi localization [162,163]. The physiological stimuli that regulate the translocation to the plasma membrane are not known.

The transporters most frequently overexpressed in cancer are GLUT1 and GLUT3. GLUT1 is ubiquitous but is expressed at the highest levels in red blood cells [105], brain endothelial cells where it transports glucose across the blood-brain barrier [106], glial cells [107], and blastocyst [108]. GLUT3 is the neuronal glucose transporter [107,118], and is also expressed during early embryonic development, in the trophoblast lineage [108]. Both GLUT1 and GLUT3 have high affinity for glucose, 2-deoxyglucose, and 2-[^18^F] fluoro-deoxyglucose (FDG) [164,165]. For this reason, positron emission tomography (PET) measuring FDG uptake in vivo is used effectively for staging of most cancers [166,167,168]. GLUT1 was the first member of the family to be discovered [169] and is the most frequently expressed in cancer [165,170,171]. GLUT1 is upregulated in cancer by the following oncogenes: SRC [172], RAS [173], MYC [174], and AKT [175]. GLUT1 activation by growth factors and oncogenic stimuli is mediated by transcriptional activation [176]. Tumor suppressor p53 inhibits GLUT1 expression [177] and oncogenic p53 mutation induces GLUT1 translocation to the plasma membrane [178]. GLUT1 translocation is also negatively regulated by thioredoxin-interacting protein (TXNIP); inhibitory phosphorylation of TXNIP induced either by metabolic stress through AMPK or by oncogenic stimuli through AKT releases the intracellular GLUT1 and increases glucose uptake [179,180]. In addition, GLUT1 is regulated in the tumor microenvironment by hypoxia; its transcription is directly stimulated by HIF1 [74,181]. GLUT3 is upregulated in tumors by the oncogenic stimuli: mammalian target of rapamycin [182], epidermal growth factor receptor [183], β-catenin [184], and epithelial to mesenchymal transition [185]. Similar to GLUT1, GLUT3 is also directly induced by HIF1 [186] and by p53 deletion [187]. GLUT14 has 95% homology with GLUT3 but its role in physiology or cancer is not known [130].

GLUT2 is expressed in the basolateral membrane of intestine and kidney cells where it is responsible for cellular export of glucose into the bloodstream [111,112]. It has also been involved in glucose sensing in pancreatic beta cells [114]. Its kinetics with low affinity is adapted for glucose and fructose export and sensing, rather than uptake in epithelial cells. Its expression has been reported in breast, colon, liver, and lung cancer [188,189,190]. GLUT4 is the insulin-regulated member of the family [125,126], expressed in the following insulin-responsive tissues: skeletal muscle, adipose tissue, and heart [122]. GLUT4 expression has been reported in multiple myeloma [191], ovarian [192] and gastric cancer [193]. Similar to GLUT1, GLUT4 is repressed by the tumor suppressor p53 [177].

The role of class II and III GLUTs in cancer is less characterized than that of class I transporters. GLUT5 does not transport glucose but is a fructose transporter expressed in intestine and kidney [128]. GLUT9 is considered to be mainly a uric acid transporter [131]. GLUT6 is expressed in endometrial cancer and is required for glycolytic reprograming and cancer cell survival [194]. GLUT8 and GLUT11 have been reported to be upregulated in multiple myeloma and required for cell survival [191]. GLUT8 is also expressed in endometrial cancer [195]. GLUT8 is required to fuel serine biosynthesis in a subset of KRAS/NRF2 mutant lung cancer cell lines [196].

### 4.2. SGLTs in Cancer

The sodium-glucose transporters belong to the SLC5A family of sodium-driven solute carriers, which includes 12 transporters with different substrate specificities. In addition to SGLT1, SGLT2, SGLT4 and SGLT5, which are hexose transporters, and SGLT3, which is a glucose sensor, it includes transporters for myoinositol (SLC5A3 and SLC5A11), monocarboxylates (SLC5A8 and SLC5A9), iodide (SLC5A5), multi-vitamin (SLC5A6), and choline (SLC5A7). Structurally, the best characterized SGLT, SGLT1 and SGLT2, have 14 transmembrane domains with both N- and C-termini on the extracellular side, an extracellular N-linked glycosylation site, and a long intracellular loop between helices 13 and 14 [197]. SGLT1 is expressed in the brush border membrane of the small intestine epithelium, where it is responsible for absorption of glucose [151,152]. It is also present in the S3 segment of the proximal convoluted kidney tubule, where it contributes to glucose re-absorption [197]. SGLT2 expression is mostly restricted to the apical membrane of the S1 and S2 segments of the proximal convoluted kidney tubule, where it is responsible for reabsorption of the majority of the filtrated glucose [151,155]. Both in the intestine and in the kidney, the glucose imported by SGLT1 and SGLT2 is, then, exported by GLUT2 expressed on the basolateral membrane [197]. Recently, SGLT2 has been reported to have a glucose-sensing function in pancreatic alpha cells, where it regulates the secretion of glucagon [156,198]. SGLT3 can transport sodium but not glucose, therefore, it is considered to be a glucose sensor expressed in the cholinergic neurons of the intestinal submucosal and myenteric plexuses [158]. SGLT4 and SGLT5 are not well characterized, but appear to be responsible for absorption/reabsorption of other hexoses, i.e., mannose [159] and fructose [160], respectively.

SGLT1 has been reported to be expressed in pancreatic [2,199], prostatic [2], ovarian [200], colorectal [201], and head and neck [202] carcinomas. Expression of SGLT1 in cancer is regulated by oncogenic epithelial growth factor receptor (EGFR) by direct, ligand-independent interaction and stabilization of the glucose transporter [203]. SGLT2 has been reported to be expressed in pancreatic [2], prostatic [2], and lung [3,204] carcinomas and in high-grade astrocytomas [205]. The mechanism of upregulation of SGLT2 in cancer is currently unknown. However, a link between transporter expression and cellular differentiation has been recently reported in lung cancer, as discussed in the next section.

### 4.3. Glucose Transporters in Lung Cancer

Lung cancer is a heterogeneous group of diseases, according to the cell of origin and the state of differentiation [206,207]. The most frequent histological types are adenocarcinoma and squamous cell carcinoma [208]. Squamous cell carcinoma (SqCC) arises from basal cells of the tracheal and bronchial epithelium [209], whereas adenocarcinoma (ADC) develops in the distal airways and the cell of origin is thought to be type II alveolar cells or bronchoalveolar stem cells [210,211]. These two cancer types present distinct metabolic phenotypes with different expression patterns of two major transporters, i.e., GLUT1 and SGLT2.

Although multiple glucose transporters have been reported to be expressed in the lung, neither GLUT1 nor SGLT2 are expressed physiologically in the cells of origin of SqCC and ADC. GLUT1 is expressed during lung development [212], but in the adult lung it is not expressed in the bronchial [213] nor in the alveolar epithelium [3]. SGLT2 expression is physiologically restricted to the kidney [151]. SGLT1 has been reported to be expressed in type II alveolar cells [151], but its expression in lung cancer is unknown.

**Figure 5:**
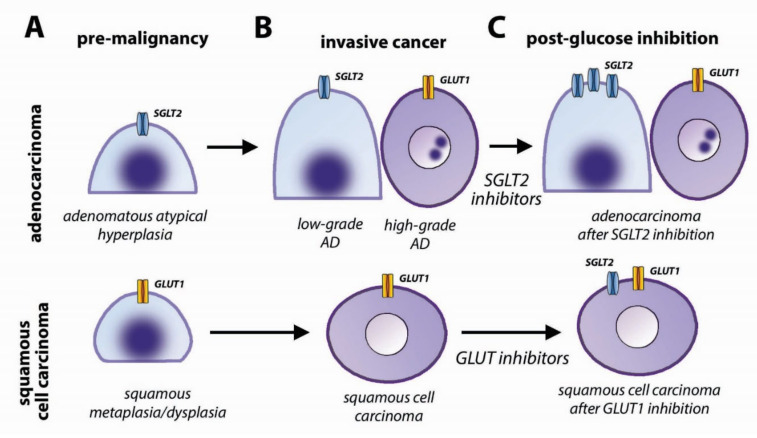
Heterogeneity of glucose transport in lung cancer. Schematic representation of glucose transporter expression in the two most frequent types of lung cancer: adenocarcinoma (upper panels) and squamous cell carcinoma (lower panels). The figure presents the major glucose transporters expressed in different stages of cancer development. (**A**) premalignant lesions; (**B**) Invasive cancer; and (**C**) invasive cancer after therapy with glucose transport inhibitors.

SqCC is characterized by high expression of GLUT1 in almost all cases [214,215,216,217], from the premalignant lesions of bronchial epithelium [213] to metastatic cancer (Figure 5A,B). GLUT1 is upregulated in SqCC by p63, the lineage factor of basal bronchial epithelial cells, and Sox2 [5], and is associated with SqCC vulnerability to glucose restriction by pharmacological inhibition of GLUT1 [4]. GLUT3 has been less studied but has shown a similar distribution to GLUT1, being associated with squamous cell histology, poorly differentiated lung cancer, and poor prognosis [218,219,220].

**Figure 6:**
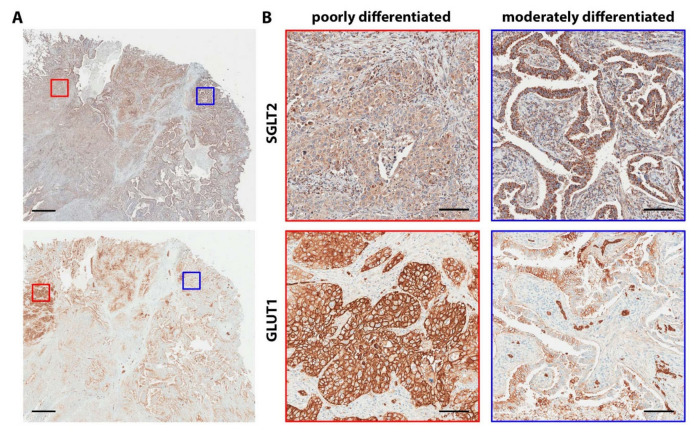
Glucose transport heterogeneity in lung adenocarcinoma. A representative sample of human lung adenocarcinoma was stained with specific antibodies against SGLT2 (upper panels) and GLUT1 (lower panels) in adjacent sections. (**A**) Low magnification showing heterogeneous distribution of the SGLT2 and GLUT1 expression in the tissue. Scalebar, 1 mm; (**B**) Higher magnifications of the areas delimited by the red and blue squares in A. The red square highlights an area of poorly-differentiated GLUT1-positive cancer, the blue square delimits a region of moderately-differentiated SGLT2-positive cancer. Scalebars, 100 µm.

In lung adenocarcinomas, glucose transporter expression evolves during the progression from premalignant lesions to invasive cancers. Premalignant lesions characterized by a lepidic-pattern growth are atypical adenomatous hyperplasia, adenocarcinoma in situ, and minimally invasive adenocarcinoma. These lesions are typically negative for GLUT1 and positive for SGLT2. As tumors become invasive and de-differentiate, they switch from a SGLT2-positive to a GLUT1-positive phenotype (Figure 5A,B) [3]. Since adenocarcinomas are typically heterogeneous, with areas of well-, moderately-, and poorly-differentiated cancer nests coexisting in the same tumor mass, also glucose transporter expression is heterogeneous, with the well-differentiated areas of the tumors expressing SGLT2 and the poorly-differentiated areas expressing GLUT1 (Figure 6) [3]. This pattern is consistent with previous reports that GLUT1 is expressed at higher levels in poorly-differentiated than in well-differentiated lung tumors [215,217,221], and that in lung adenocarcinoma, high GLUT1 and high FDG uptake are correlated with solid (poorly differentiated) morphology and worse prognosis [216,217,222,223,224].

### 4.4. Clinical Implications

The biological meaning of glucose transport heterogeneity in lung cancer is currently unknown, but it suggests that different glucose transporters are associated with distinct metabolic phenotypes, with important clinical implications. Since glucose transport is a limiting step in cancer metabolism [1,100,101,102], glucose transport inhibition is emerging as a novel treatment for lung and other cancers. GLUT1 inhibitors and SGLT2 inhibitors have shown efficacy in reducing cancer growth and prolonging survival in mouse models of SqCC and ADC, respectively [3,4]. However, glucose transport inhibition induces adaptations; SqCCs upregulate SGLT2 after GLUT1 inhibition [4], whereas ADCs upregulate SGLT2 under SGLT2 inhibition treatment [3] (Figure 5C). The heterogeneity and plasticity of glucose transporter expression in lung cancer is likely to hinder the response to glucose transport inhibitors and highlights the need for a thorough characterization of the basal metabolic phenotype and the metabolic adaptations to therapies. GLUT activity can be measured in vivo by positron emission tomography (PET) with the tracer 2-[^18^F] fluoro-deoxyglucose (FDG) [165]. FDG PET is a very sensitive tool for whole-body detection of cancer metabolic activity [167,225]. However, FDG has low affinity for SGLTs [226]; consistently, early lesions of the lung adenocarcinoma spectrum, which do not express GLUT1, typically show low FDG uptake [227,228]. Another tracer, methyl-4-[^18^F] fluoro-deoxyglucose (Me4FDG), is transported by SGLTs and not by GLUTs [2,229,230], and can be used to image early lung adenocarcinomas that are FDG-negative [2]. Multitracer and longitudinal imaging with both FDG and Me4FDG can give a complete picture of glucose uptake in cancer, and of the metabolic adaptations induced by therapy. We anticipate that in the future a multitracer characterization of the metabolic phenotype of tumors will be able to guide metabolic treatments.

Considering the metabolic heterogeneity and plasticity of cancer cells, glucose transport inhibition is expected to induce adaptation and resistance in lung cancer [3,4]. Although the efficacy of blocking a single transporter is limited in cancer cells that can upregulate multiple transporters, glucose inhibition can sensitize cancer cells to other therapies, such as mitochondria targeting agents [231,232]. Another possibility is to identify subsets of cancers that are exquisitely sensitive to glucose deprivation, such as the double mutants of KRAS and KEAP1, which have been shown to be dependent on GLUT8 activity [196].

Another factor to consider for cancer metabolic treatments is the potential adverse drug reactions induced by blocking glucose transport in normal tissues. SGLT2 inhibitors have been used extensively in clinical trials and in clinical practice, because SGLT2 is the major transporter responsible for glucose reabsorption in the kidney proximal tubules [197]. Therefore, SGLT2 inhibition is an effective treatment for type II diabetes mellitus with a good safety profile [233,234]. Major side effects have been increased risk for urinary tract infections, infrequent hypoglycemia, and diabetic ketoacidosis [233]. These side effects are likely related to the underlying pathophysiology of diabetes and are not likely to occur in nondiabetic patients. Less is known about GLUT inhibitors, which have been studied in murine models but not in patients [4,235]. While no major adverse reactions have been reported in mice, an important concern for human studies are the potential neurologic and hematologic effects related to the inhibition of GLUT1 in the endothelial cells of the blood-brain barrier, which could compromise the glucose supply to the brain [109,110], and in erythrocytes, which depend on GLUT1 activity for glucose uptake [105]. The key in clinical translation of GLUT inhibitor treatments involves the following: (i) the identification of a therapeutic window exploiting the increased dependence of cancer cells on glucose as compared with normal tissues and (ii) the development of specific inhibitors that can target alternative transporters with less prominent physiological functions than GLUT1, such as class III GLUTs. The role of these transporters in cancer is just starting to be unveiled [194,196].

## 5. Concluding Remarks

Since the first observations by Warburg a century ago, we have learned a lot about cancer metabolism, glucose addiction, and metabolic vulnerabilities of cancer cells. The next challenge is to address the heterogeneity of cancer metabolism. The following information is now clear: (1) cancer cells can express multiple and redundant nutrient transporters, unlike normal cells; (2) not all the cells in a cancer tissue have the same metabolic phenotype; and (3) normal cells cooperate and compete with cancer cells in the tumor microenvironment. Therefore, any therapeutic intervention applies a selective pressure that changes the complex equilibrium of metabolic interactions in the tumor and eventually induces mechanisms of cancer cell adaptation and resistance. Methods to track the evolution of metabolic phenotypes in vivo, such as PET imaging, would help clinicians to characterize these adaptations and target them with personalized treatment strategies.

## Figures and Tables

**Table 1:** Physiological properties of glucose transporters.

Transporter	Km for Glucose	Other Substrates	Expression in Normal Tissues	KO Phenotype	Notes
**Class I GLUTs**					
GLUT1 (SLC2A1)	3 mM [103,104]	Galactose, mannose, glucosamine	Red blood cells [105]; Blood-brain barrier [106]; Glial cells [107]; Early embryonic development [108]	-/- embryonic lethal +/- seizures, developmental delay, microcephaly, ataxia [109,110]	
GLUT2 (SLC2A2)	17 mM [103]	Glucosamine [103]	Small intestine (basolateral [111]), kidney tubules (basolateral [112]), liver [113]; Pancreatic beta-cells [114]	-/- type 2 diabetes mellitus, neonatal death [115]	
GLUT3 (SLC2A3)	1.4 mM [116]	Xylose, mannose [117]	Neurons [107,118]; Early embryonic development [108]	-/- embryonic lethal [119]; +/- features of autism spectrum [120]	
GLUT4 (SLC2A4)	4 mM [121]	Dehydroascorbic acid, glucosamine	Skeletal muscle, adipose tissue, heart [122]	-/- growth retardation, cardiomegaly [123]; +/- diabetes [124]	Insulin-dependent translocation [125,126]
GLUT14 (SLC2A14)	?	?	Testis [127]	?	95% homology with GLUT3
**Class II GLUTs**					
GLUT5 (SLC2A5)	n/a	Fructose [128]	Small intestine, Kidney, testes [128]	-/- fructose malabsorption [129]	
GLUT7 (SLC2A7)	0.3 mM [130]	Fructose [130]	Small intestine, colon [130]	?	68% homology with GLUT5 [130]
GLUT9 (SLC2A9)	0.6 mM [131]	Fructose [131], uric acid [131]	Kidney tubule, liver [132]; Pancreatic beta cells [133]	-/- hyperuricemia, urate nephropathy [134]	
GLUT11 (SLC2A11)	0.1 mM [131]	Fructose [131]	Heart, skeletal muscle, adipose tissue, kidney, pancreas [135]	?	
**Class III GLUTs**					
GLUT6 (SLC2A6)	17.5 mM (zebrafish) [136]	?	Brain, spleen, leukocytes [137]; Intracellular (lysosomal) [138]	-/- minimal effects (reduced fat in female mice) [139]	Previously known as GLUT9 [137]
GLUT8 (SLC2A8)	2 mM [140]	?	Testis, brain; Intracellular [140]; lysosomal [141]	-/- hyperactivity [142]	Previously known as GLUTX1
GLUT10 (SLC2A10)	0.3 mM [143]	Galactose [143]	Heart, lung [143]	Mutants: thickened, irregular arteries [144]	
GLUT12 (SLC2A12)	?	Galactose, fructose [145]	Heart, skeletal muscle, prostate, adipose tissue, small intestine [146]	Knock-down in zebrafish: impaired cardiac development, arrhythmias; hyperinsulinemia, insulin resistance [147]	Insulin-induced translocation [146]
GLUT13 (SLC2A13)	n/a	Myoinositol; Inositol-3-phosphate [148]	Brain [148]	?	
**SGLTs**					
SGLT1 (SLC5A1)	0.3 mM [149,150]	Galactose, α-methyl-deoxyglucose	Small intestine (apical), kidney proximal tubule, heart, liver, lung, pancreatic ducts, prostate, salivary glands [151,152]	-/- glucose-galactose malabsorption [153]	
SGLT2 (SLC5A2)	6 mM [149,150,154]	α-Methyl-deoxyglucose [154]	Kidney proximal tubule (apical) [151,155], pancreatic ducts [2], pancreatic alpha cells [156]	-/- glycosyuria [157]	
SGLT3 (SLC5A4)	60 mM [158]	α-Methyl-deoxyglucose [158]	Small intestine, skeletal muscle [158]	?	For the low affinity, it acts as a glucose sensor, not transporter, at physiological sugar concentration and pH
SGLT4 (SLC5A9)	1.6 mM [159]	Mannose [159]	Small intestine, kidney, liver [159]	?	
SGLT5 (SLC5A10)	10 mM [160]	Mannose > fructose > glucose > galactose [160]	Kidney [160]	-/- fructosuria, hepatic steatosis [161]	

n/a—there is no Km value because the transporter does not transport glucose; ?—unknown; -/- Homozygous knockout; +/- Heterozygous knockout;
